# Combination Treatment With Metformin and Tacrolimus Improves Systemic Immune Cellular Homeostasis by Modulating Treg and Th17 Imbalance

**DOI:** 10.3389/fimmu.2020.581728

**Published:** 2021-01-08

**Authors:** Soon Kyu Lee, Min-Jung Park, Joo Yeon Jhun, Jin-Ah Beak, Jeong Won Choi, Jae-Yoon Rye, Jeong Won Jang, Si Hyun Bae, Seung Kew Yoon, Ho Joong Choi, Young Kyoung You, Mi-La Cho, Jong Young Choi

**Affiliations:** ^1^ Division of Hepatology, Department of Internal Medicine, Seoul St. Mary’s Hospital, College of Medicine, The Catholic University of Korea, Seoul, South Korea; ^2^ The Rheumatism Research Center, Catholic Research Institute of Medical Science, College of Medicine, The Catholic University of Korea, Seoul, South Korea; ^3^ Division of Hepatology, Department of Internal Medicine, Eunpyeong St. Mary’s Hospital, College of Medicine, The Catholic University of Korea, Seoul, South Korea; ^4^ Department of Surgery, Seoul St. Mary’s Hospital, College of Medicine, The Catholic University of Korea, Seoul, South Korea

**Keywords:** metformin, tacrolimus, regulatory T cell, Th17 cell, allogeneic response, GVHD, liver transplantation

## Abstract

We examined the effect of combination therapy with metformin and tacrolimus on immune parameters including T regulatory (Treg) and type 17 helper T (Th17) cells *in vitro* and *in vivo* in mice and in liver transplantation (LT) patients. T cell proliferation and subtypes after *in vitro* T cell activation or allogeneic stimulation were evaluated. RNA sequencing and microarray analysis were used to evaluate differences in gene expression. Metformin and tacrolimus were administered to mice with graft-versus-host disease (GVHD) and the effects *in vivo* were assessed. Five LT patients were treated with metformin and the changes in Treg and Th17 cells examined. Combination therapy decreased Type 1 helper T (Th1) and Th17 cells present after *in vitro* T cell activation, whereas genes associated with Treg were overexpressed. During *in vitro* allogeneic stimulation, combination therapy increased Treg cells and decreased T cell proliferation and pro-inflammatory markers. In mice with GVHD, combination treatment decreased the clinical and pathological severity of GVHD. In LT patients, addition of metformin increased the peripheral percentage of CD4+Treg and CD8+Treg cells and decreased CD4+Th17. Our study suggests that the addition of metformin to tacrolimus may improve immunological balance by increasing Treg cells and decreasing Th17 cells.

## Introduction

Tacrolimus, a calcineurin inhibitor (CNI), is one of the main immunosuppressive drugs used to prevent rejection after liver transplantation (LT) (1, 2). It has immunosuppressive effects by reducing interleukin-2 (IL-2) transcription, which results in inhibition of the proliferation of helper and cytotoxic T cells (2). However, a limitation of tacrolimus is that it also reduces the levels of CD4^+^CD25^+^FOXP3^+^ regulatory T (Treg) cells, which are crucial for maintaining tolerance (3). Indeed, in our previous study, the serial increase of Treg/type 17 helper T (Th17) ratio during tapering immunosuppressive drugs may indicate tolerance after LT (4).

Metformin is a well-known antidiabetic drug that also has anticancer effects by increasing CD8+T cells (5, 6). Moreover, metformin can modulate the immune response by activation of the energy sensor 5′-AMP-activated protein kinase (AMPK), and inhibition of mammalian target of rapamycin (mTOR) and signal transducer and activator of transcription 3 (STAT3) (7). As a result, metformin modulates the immune system by reducing type 17 helper T (Th17) cells and increasing the Treg population (7).

To compensate for the immune imbalance caused by tacrolimus, combinations of IL-2 therapy, mycophenolate mofetil (MMF), or mTOR inhibitors have been used in attempts to increase Treg cells (8–10). Considering the immune pathways modulated by metformin, the combination of metformin with tacrolimus may have beneficial immune effects by balancing Treg/Th17 cells. However, combination treatment with metformin and tacrolimus has not been studied in LT recipients.

In this study, we performed *in vitro* and *in vivo* analyses to examine the immune modulating effect of combination therapy with metformin and tacrolimus after T cell activation and in a mouse model of graft-versus-host disease (GVHD). Moreover, we also performed a preliminary evaluation of the effect of combination therapy on the balance of Treg/Th17 in LT patients.

## Methods and Materials

### Mice

Balb/c (B/c) and C57BL/6 (B6) mice, 8–10 weeks of age, were obtained from OrientBio (Sungnam, Korea) and were kept under specific-pathogen-free conditions in an animal facility. A HEPA filter system was used to exclude bacteria and viruses from the air in the facility. The protocols used in our study were approved by the Animal Care and Use Committee of the Catholic University of Korea (CUMC-2018-0036-05).

### Patients

LT patients with newly developed diabetes mellitus (DM) were recruited in Seoul St. Mary’s Hospital. The inclusion criteria were: more than 18 years old, patients being treated with CNIs including cyclosporine or tacrolimus, and patients who had received LT more than 3 years previously and had not experienced rejection. Five patients were included and evaluated for Th17 and Treg levels before and after 3 months of metformin treatment (1000 mg/day). This study was approved by the Institutional Review Board of the Catholic University of Korea (KC18EESI0363).

### Murine and Human T Cell Isolation and T Cell Activation

To purify mouse splenic or human peripheral blood CD4+T cells, splenocytes or peripheral blood mononuclear cells were incubated with CD4-coated magnetic beads and isolated using magnetic-activated cell sorting separation columns (Miltenyi Biotec, Bergisch Gladbach, Germany). Isolated CD4+T cells were activated by treatment with anti-CD3 (0.5 µg/ml), and soluble anti-CD28 (0.5 µg/ml) for 3 days in complete culture medium (RPMI 1640 supplemented with 10% [v/v] heat-inactivated fetal calf serum).

### Conditioning of Murine and Human Alloreactive T Cell Responses

Splenocytes derived from B6 mice were used as “stimulator” cells in the context of allorecognition. Cells from B/c mice were used as the “responder” cells in this assay. Splenocytes were harvested in ACK lysis buffer (0.15 M NH4CL, 10 mM KHCO_3_, and 0.1 mM EDTA; pH 7.2–7.4), washed, and resuspended in complete culture medium (RPMI 1640 supplemented with 10% [v/v] heat-inactivated fetal calf serum). Aliquots of 2 × 10^5^ CD4^+^T cells (responders) were cultured with 2 × 10^5^ irradiated (2,500 cGy) stimulators in 96-well plates containing 200 µl/well of complete medium, at 37°C in a humidified 5% (v/v) CO2/air atmosphere. To observe the human alloreactive T cell response, peripheral blood mononuclear cells (PBMCs) were isolated from two healthy volunteers were examined. Human responder CD4+T cell (2×10^5^) were irradiated with 5000 cGy and seeded with stimulator PBMCs (2×10^5^) into 96-well plates for 4 days.

Mouse and human alloreactive T cell proliferation was measured by tritiated thymidine (3[H]-TdR) uptake. The cells were pulsed with 1 µCi of (3[H]-TdR (NEN Life Science Products, Boston, MA, USA) 18 h before harvesting using an automated harvester (PHD Cell Harvester; Cambridge Technology, Cambridge, MA, USA) and counted in a β-counter (Packard TopCount NXT).

### GVHD Model

Recipient (B/c) mice were lethally irradiated with 700 cGy, then intravenously injected with donor (B6) bone marrow cells (5 × 10^6^) and splenocytes (5 × 10^6^) (to induce acute GVHD). All experiments were performed at least three times, with six mice per group. After the induction of GvHD, recipient mice were administered Met (50 mg/kg) and tacrolimus (10 mg/kg) every 2 days *via* intraperitoneal injection. Control GvHD mice were administered vehicle (dimethyl sulfoxide diluted in saline) *via* the same manner as the treatment group.

Survival after bone marrow transplant (BMT) was monitored daily and the extent of clinical GVHD assessed weekly using a scoring system that summed the changes in five clinical parameters: weight loss, posture, activity, fur texture, and skin integrity (11). Mice were euthanized on day 38 after BMT prior to blinded histopathology of GVHD target tissues (skin, liver, and the small and large intestines).

### Histological and Immunohistochemical Analyses

Mice were euthanized on day 38 after BMT and organs harvested, cryoembedded, and sectioned. Tissue specimens were fixed in 10% formalin buffer and embedded in paraffin. Sections (6 µm thick) were stained with hematoxylin and eosin and the histologic score was determined using an established scoring system (11). For immunohistochemistry staining, sections were stained with primary antibodies against interferon (IFN)-γ and IL-17 overnight at 4°C, followed by addition of a biotinylated secondary antibody and a streptavidin-peroxidase mixture for 1 h (ThermoFisher, San Diego, CA, USA). Color was developed by addition of 3,3-diaminobenzidine (Dako, Carpinteria, CA, USA).

### Flow Cytometry and Enzyme-Linked Immunosorbent Assays (ELISA)

Mononuclear cells were stained with various combinations of fluorescent antibodies against Foxp3, IFN-γ, CD4, CD8, CD25, and IL-17. Prior to intracellular staining, cells were restimulated for 4 h with phorbol myristate acetate (25 ng/ml) and ionomycin (250 ng/ml) in the presence of GolgiSTOP (BD Biosciences, San Diego, CA, USA). Intracellular staining was performed using a kit (eBioscience, Thermo Fisher Scientific, Waltham, MA, USA) following the manufacturer’s protocol. Flow cytometry was performed with the aid of a FACSCalibur instrument (BD Biosciences). The concentrations of IFN-γ and IL-17 in culture supernatants and serum were measured using a sandwich ELISA (Duoset; R&D Systems, Lille, France).

### RNA Sequencing Analysis, Microarray Data, and Kyoto Encyclopedia of Genes and Genomics (KEGG) Pathway

T cells were isolated from wild-type (WT) mice and treated in T cell activation conditions with or without metformin and tacrolimus. RNA sequencing analysis using next-generation sequencing was used to document the existence and amount of mRNA. Affymetrix microarrays HT_MG-430A were used to measure the resulting mRNA. Expression data was preprocessed using the range migration algorithm followed by quantile normalization. KEGG pathway was used to represent the molecular interaction and expression of gene pathway.

### Statistical Analysis

Values are presented as means ± standard deviation. Analyses of differences between groups were performed using unpaired Student’s *t*-test or Mann-Whitney *U* test and one-way analysis of variance (ANOVA) where appropriate. Statistical analysis was performed using IBM SPSS Statistics for Windows (v. 24; IBM Corp., Armonk, NY, USA).

## Results

### Modulation of T Cells Subtypes in Mice After Combination Treatment With Metformin and Tacrolimus

**Figure 1:**
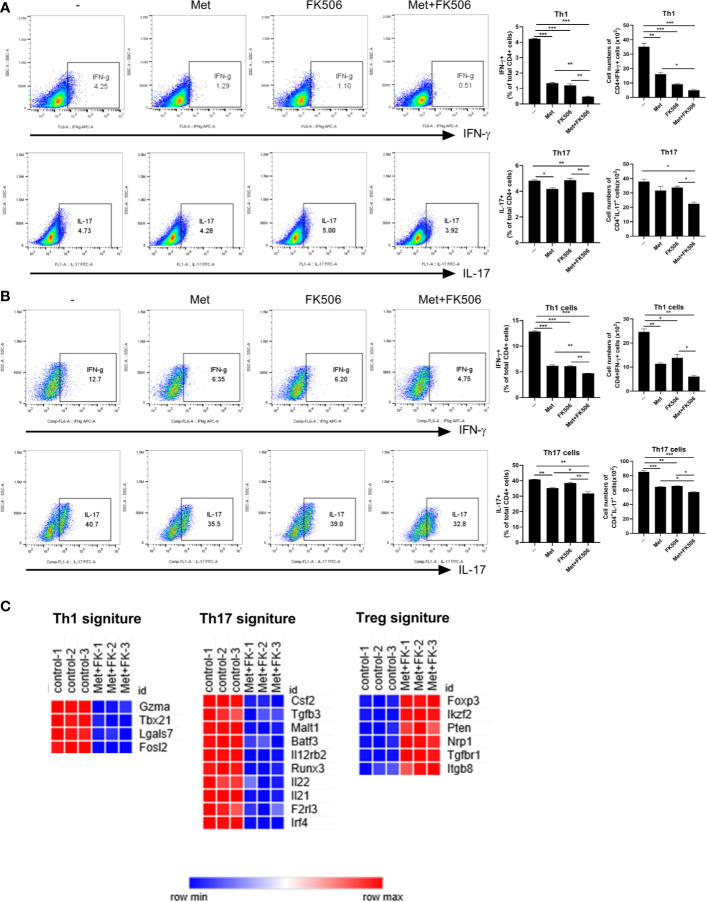
The change in T cell subtypes induced by combination therapy with metformin and tacrolimus. **(A)** The proportion of Th1 and Th17 cells in normal mice treated with combination therapy. CD4+T cell isolated from normal C57BL/6 mice were stimulated with anti-CD3 (0.5 ug/ml) in the presence of Metformin or FK506 alone or combined Metformin and FK506 for 3 days and analyzed by flow cytometry. A plot from one representative experiment displays the proportions of IL-17+, IFN-γ + among CD4+ T cells. **(B)** The difference in proportions of Th1 and Th17 cells in mice with graft-versus-host disease after combination therapy. CD4+T cell isolated from GVHD mice were stimulated with anti-CD3 (0.5 ug/ml) in the presence of Metformin or FK506 alone or combined Metformin and FK506 for 3 days and analyzed by flow cytometry. A plot from one representative experiment displays the proportions of IL-17+, IFN-γ + among CD4+ T cells. Numbers in the plots indicate percentages of gated cells. Data are means ± SEMs. Data are representative of three independent experiments. (*p < 0.05, **p < 0.01, ***p < 0.005) **(C)** Gene expression analysis by RNA sequencing before and after treatment with combination therapy. CD4+T cell isolated from normal C57BL/6 mice were stimulated with anti-CD3 (0.5 ug/ml) in the presence of Metformin or FK506 alone or combined Metformin and FK506 for 3 days and analyzed by RNA-seq data. Heatmaps of genes encoding for molecules involved in Th1, Th17 cells, and Treg cell function that are differentially expressed in combination of metformin and FK506 compared to vehicle.

We examined the changes in type 1 helper T (Th1) and Th17 cells in the CD4^+^T cells derived from splenocytes of normal and GVHD mice after CD3 stimulation. In normal mouse cells activated under CD3 stimulation, flow cytometry demonstrated that the combination of metformin and tacrolimus suppressed the development of Th1 and Th17 cells compared with control and tacrolimus monotherapy (**Figure 1A**). Similarly, in activated T cells from GVHD mice, development of Th1 and Th17 cells was suppressed by combination therapy with metformin and tacrolimus (**Figure 1B**).

In hierarchical clustering analysis of the patterns of expression and KEGG pathway, combination therapy was similar to tacrolimus with some change of the expression collaborated with metformin combination (**Supplementary Figures 1A, B**). Combination therapy enhanced the expression compared to metformin monotherapy (**Supplementary Figures 2A, B**). RNA sequencing analysis to evaluate the differences in gene expression revealed decreased expression of gene signatures related to Th1 and Th17 cells in combination therapy. In contrast, expression of genes related to Tregs, including Foxp3, was more increased after combination treatment with metformin and tacrolimus (**Figure 1C**).

Furthermore, expression of gene signatures related to glycolysis and Th17 related gene decreased in combination therapy compared to metformin monotherapy, whereas expression of genes related Tregs increased more after combination treatment with metformin and tacrolimus than metformin monotherapy (**Supplementary Figure 2C**).

### Suppression of Alloreactive T Cells by Combination Treatment With Metformin and Tacrolimus

**Figure 2:**
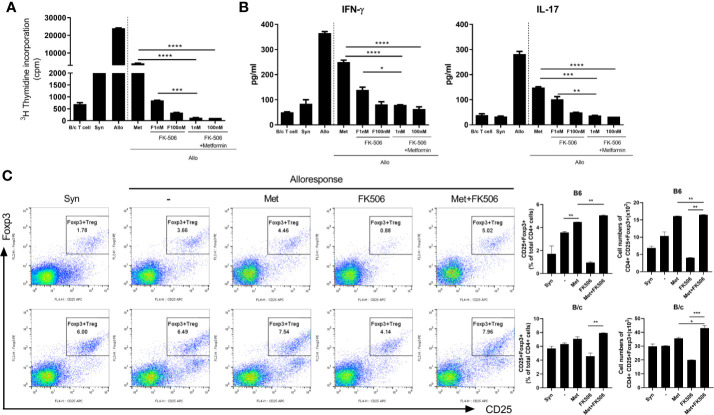
The suppressive effect of metformin–tacrolimus combination treatment on alloreactive T cell responses. **(A)** The difference in proliferation of alloreactive T cells in each treatment. In the mixed lymphocyte reaction assay, a total of 105 B/c splenic T cells (responders) were incubated with 105 irradiated B/c (syngeneic stimulators, Syn) or B6 (allogeneic stimulators, Allo) splenic APCs for 4 days. Responder cells were cultured in the presence or absence of metformin or/and FK506. **(B)** The suppression of pro-inflammatory markers after treatment with combination therapy. IFN-γ and IL-17 levels in the supernatants were measured by ELISA. **(C)** The activity of alloresponsive Treg cells after each treatment. Foxp3+ Treg cells was performed were determined by flow cytometry. (*p < 0.05, **p < 0.01 ***p < 0.005, ****p < 0.001).

After *in vitro* allogeneic stimulation of mouse cells, the proliferation of alloresponsive T cells and the level of pro-inflammatory markers were compared between groups treated with metformin monotherapy (500 uM), tacrolimus monotherapy (1 nM, 100 nM), or a combination of both drugs. The combination of metformin with tacrolimus decreased the proliferation of alloresponsive T cells to a greater extent than metformin or tacrolimus monotherapy in mice (*P* < 0.05, **Figure 2A**). Moreover, combined treatment significantly decreased the level of pro-inflammatory markers such as IFN-γ and IL-17 compared with monotherapy (*P* < 0.05, **Figure 2B**).

The activity of Treg cells after 4 days of allostimulation in the presence of each drug was evaluated. The population of Treg cells was significantly much higher in the presence of combination therapy than with monotherapy, especially tacrolimus (**Figure 2C**).

### Combination of Metformin and Tacrolimus Reduces the Severity of GVHD

**Figure 3:**
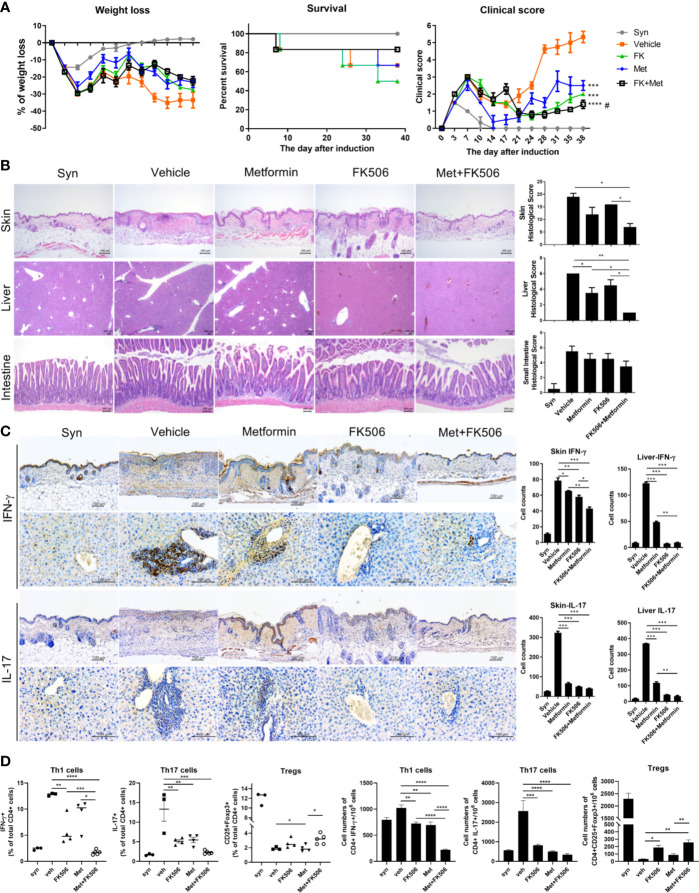
The severity of graft-versus-host disease (GVHD) after combination therapy. **(A)** The serial changes in GVHD clinical score after metformin and tacrolimus combination therapy. Splenocytes (5×10^6^) plus bone marrow cells (5×10^6^) from B6 mice were transplanted into irradiated B/c mice *via* intra-vain injection. Weight, weight change, and clinical score were monitored in mice with a GVHD. Combined data from 2 independent experiments (n=15 per group) are displayed. **(B)** Histological difference in GVHD in target organs including skin, liver, and intestine. Histopathological analysis of the skin, liver, and intestine after BMT. The sections were stained with hematoxylin and eosin (original magnification, × 100). **(C)** The expression of pro-inflammatory markers in target organs after treatment with each drug. Immuno-histochemical staining was performed to measure the expression of IL-17 and IFN-g in skin and liver tissue from each groups (scale bar, 100 μM). The positive cells for each antibody are shown at the lower panels. Data represent the mean ± SEM of 3 independent experiments **(D)** The ability to control Th1, Th17, and increase Treg cells with combination therapy were determined by flow cytometry. Data are means ± SEMs. Data are representative of three independent experiments. (*p < 0.05, **p < 0.01, ***p < 0.005, ****p < 0.001 versus vehicle treated groups, #p<0.05 versus metformin treated group).

In GVHD model mice, the survival rate and serial changes in the clinical score representing the severity of GVHD were compared between each therapy, including combination treatment. After induction, the clinical score was continuously lower with metformin or tacrolimus monotherapy compared with the control group. Moreover, it was decreased to an even greater extent with combination therapy with metformin and tacrolimus, showing a synergistic effect of this combination. Meanwhile, weight loss was partially lesser in the combination group than in monotherapy. The combination therapy group also showed a better survival rate than both the metformin and tacrolimus monotherapy groups (**Figure 3A**).

After euthanizing the GVHD model mice on day 38 after BMT, the histopathology of GVHD target tissues (skin, liver, and the small and large intestine) were analyzed. The histopathology also demonstrated that inflammatory cells and tissue damage was suppressed by combination treatment with metformin and tacrolimus (**Figure 3B**). Moreover, the expression of IFN-γ and IL-17 in the target tissues was significantly decreased after combination therapy compared with either monotherapy (**Figure 3C**).

The proportion of Treg cells in CD4^+^T cells isolated from the splenocytes of GVHD mice was significantly increased after metformin and tacrolimus combination therapy, whereas Th1 and Th17 cells were decreased after combination (**Figure 3D**).

### The Ability to Control Human Alloresponses by Addition of Metformin to Tacrolimus Therapy

**Figure 4:**
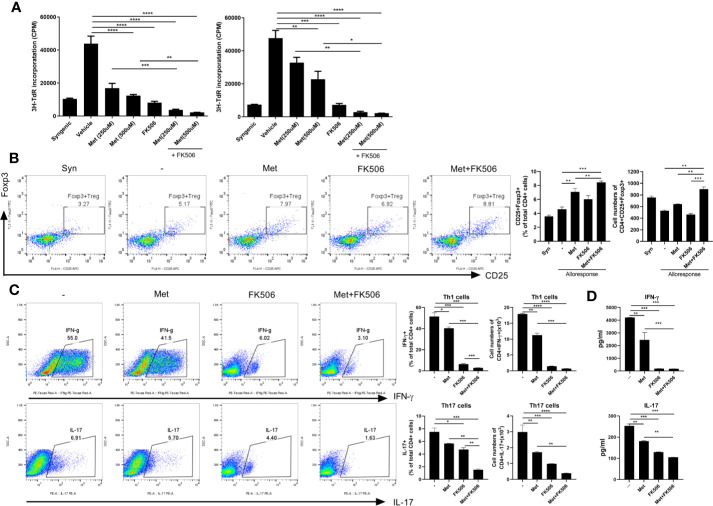
The ability to control human alloreactive T cells by metformin and tacrolimus combination treatment. **(A)** The suppression of alloreactive T cells by each treatment. CD4+ T cells from healthy donor PBMC were cocultured with APC cells from other healthy donor PBMC for 4 days in the presence or absence of metformin or/and FK506. The graph shows result from two other donors’ alloreactive T cell proliferation. **(B)** The activity of alloresponsive Treg cells after each treatment. Foxp3+ Treg cells was performed were determined by flow cytometry. **(C)** The suppression of Th1 and Th17 cells by each treatment. A plot from one representative experiment displays the proportions of IL-17+, IFN-γ + among CD4+ T cells. **(D)** The change in pro-inflammatory cytokines. IFN- γ and IL-17 levels in the supernatants were measured by ELISA. (*p < 0.05, **p < 0.01, ***p < 0.005, ****p < 0.001 versus alloresponse).

In *in vitro* stimulated human cells, the differences in proliferation of alloreactive T cells were compared between those treated with metformin (500 µM), tacrolimus (10 mM), or a combination of metformin (500 µM) and tacrolimus (10 mM). Proliferation of human alloreactive T cells was also significantly decreased by combination therapy compared with monotherapy (**Figure 4A**).

In human *in vitro* alloresponse condition, Treg cells were significantly increased in metformin monotherapy and more increased in combination therapy with metformin and tacrolimus (**Figure 4B**).

We also evaluated the population of Th1 and Th17 cells and the level of pro-inflammatory markers in allostimulated human T cells. The population of Th1 and Th17 cells was decreased by combination therapy (**Figure 4C**). Pro-inflammatory markers such as IL-17 and IFN-γ were also more profoundly decreased by metformin and tacrolimus combination therapy (**Figure 4D**).

### Improvement of the Immune Imbalance in LT Recipients Given Combination Therapy: Preliminary Data

**Figure 5:**
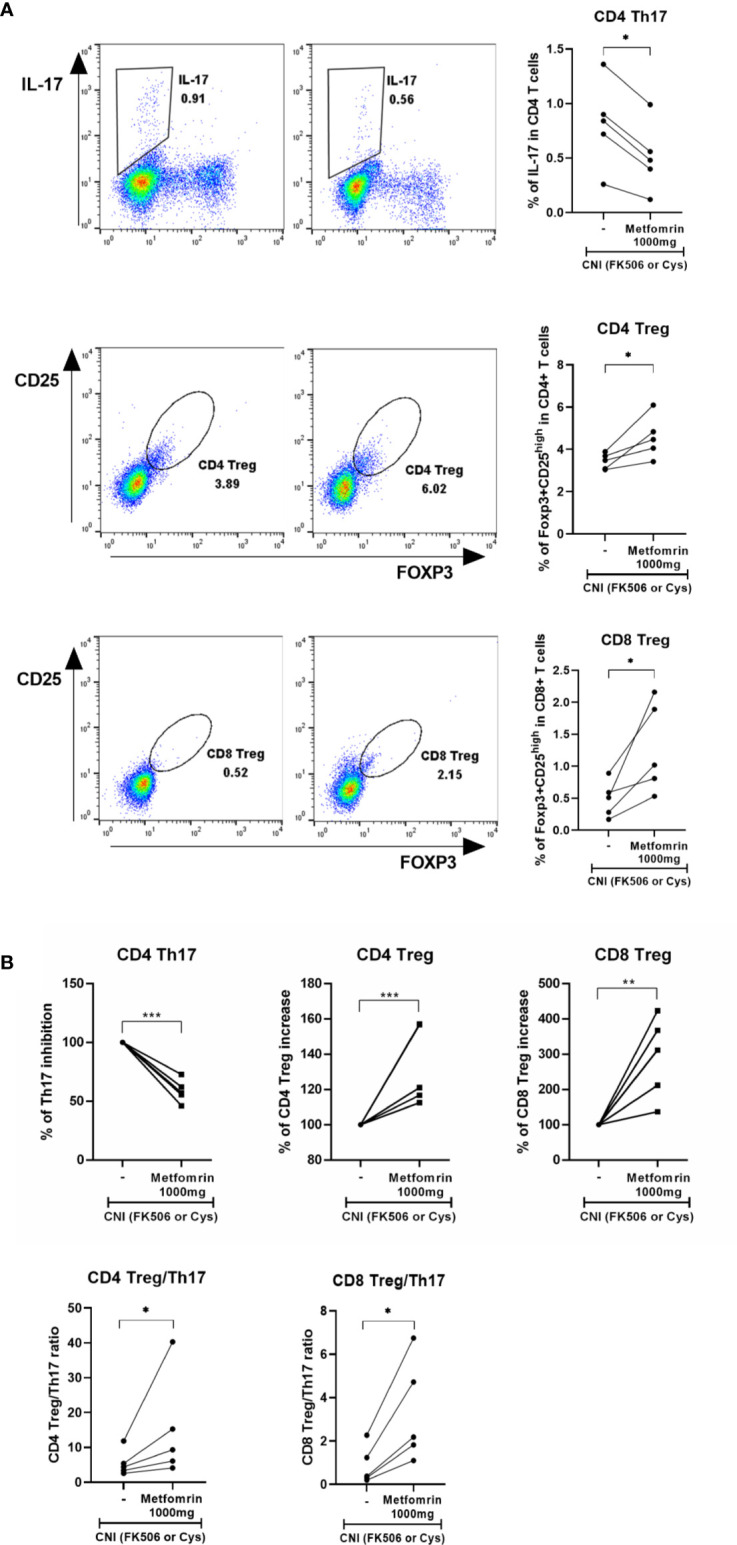
**(A, B)** Immunomodulatory effect of metformin and calcineurin inhibitor (CNI) combination treatment in liver transplant patients with diabetes. In all five patients, the population of CD4^+^Th17 cells were decreased, whereas CD4^+^Treg and CD8^+^Treg were increased after addition of metformin (1000 mg/day) treatment (*p < 0.05, **p < 0.01, *** *p* < 0.005).

We also preliminarily evaluated the changes in Treg and Th17 cells 3 months after the addition of metformin (1000 mg per day) in five LT patients treated with CNI (cyclosporine or tacrolimus). In all five patients, the population of CD4^+^Th17 cells was significantly decreased after metformin. However, the percentages of CD4^+^Treg and CD8^+^Treg cells were significantly increased after combination treatment with metformin and CNI (**Figures 5A, B**). During the metformin treatment, all five patients were tolerated without need of change in CNI dose or addition of other diabetic drugs and the blood level of markers of liver function such as aspartate transaminase and alanine transaminase were within normal range and did not change significantly (data not shown).

## Discussion

This study is the first to examine the effects of combination therapy with metformin and tacrolimus using *in vitro* and *in vivo* analysis. Metformin and tacrolimus combination therapy suppressed the proliferation of alloresponsive T cells and Th1 and Th17 cells. Combination treatment also decreased the levels of pro-inflammatory markers and increased Treg proliferation. In the mouse model of GVHD, combination therapy decreased the severity of GVHD as assessed by clinical score and histopathology. Moreover, in LT patients, our preliminary data showed that combination therapy simultaneously decreased the population of Th17 cells and increased Treg cells.

In the present study, this immunologic effect of metformin was sustained in the setting of tacrolimus treatment. Combination therapy with metformin and tacrolimus suppressed Th1 and Th17 cells, IL-17, and IFN-γ as assessed by *in vitro* and *in vivo* analyses. Metformin is a well-known antidiabetes drug that has recently been shown to have immune modulating effects *via* activation of AMPK, and inhibition of mTOR, STAT3, and PD-1 (12, 13). Several studies, including our previous study, have reported that metformin increased the population of Treg cells and decreased Th17 cells (14, 15).

Through RNA sequencing analysis, we also demonstrated that combination therapy increased expression of Foxp3, the master regulator transcription factor for development of Treg cells (16). Combination therapy may enhance the expression of Foxp3 and thereby increase the proportion of Treg cells. These results suggest that the addition of metformin may improve the Treg/Th17 balance compared with conventional tacrolimus monotherapy.

Combination therapy attenuated the severity of GVHD, both in clinical score and histopathology, to a greater extent than monotherapies. Because GVHD develops as a result of donor T cell-mediated host injury, we could easily evaluate the change in T cells caused by each treatment (17). Our results may be the result of metformin inhibiting STAT3 and promoting AMPK, as we documented previously in this GVHD model (18). The results of the present study suggest that even during tacrolimus treatment, the addition of metformin had beneficial effects on immune modulation.

Similarly to the findings of the *in vivo* and *in vitro* studies in mice, LT recipients with early DM showed an increased Treg population with decreased Th17 cells after combined treatment with CNI and metformin. We previously reported that current CNI-based treatment after LT maintained the level of effector T cells but significantly suppressed Treg cells (19). Moreover, in a recent study, an early reduction in Treg cells was also associated with acute rejection after LT in patients receiving CNI-based treatment (20). Because maintenance of Treg cells after LT is important to allow maintenance of tolerance and immune balance, we performed a preliminary study evaluating the change in Treg/Th17 cells after addition of metformin treatment in LT recipients. According to our results in LT recipients, we may expect an improvement of the Treg/Th17 balance with metformin treatment.

Our study has several limitations. First, we could not demonstrate the exact underlying mechanism of the changes after combination treatment with metformin and tacrolimus. However, as we reported previously, we could predict that the decrease in Treg cells induced by tacrolimus could be compensated by the effect of metformin in promoting AMPK and inhibiting STAT3 (18). Further study will be needed to confirm whether these mechanisms of metformin activity function during tacrolimus-based treatment. Second, we included only a small number of LT patients treated with metformin and the follow-up period was only 3 months after metformin addition. To manifest the immunologic effect of metformin in LT patients, we only included patients with early diagnosed DM who just need lifestyle modification and/or low dose of antidiabetic drugs. Our results for the LT patients are therefore preliminary, and further study is needed, with larger number of patients with long-term results for the changes in immune cellular homeostasis including Treg/Th17 cells balance. Despite these limitations, this was the first study to evaluate the possibility of using metformin as an additional immunologic control not only *in vivo* and *in vitro* but also with LT patients during tacrolimus-based treatment.

In conclusion, the findings of this study suggest that metformin could improve the immunological imbalance in tacrolimus monotherapy by increasing Treg cells and decreasing Th17 cells. Our results may suggest the possibility that the combination therapy with metformin and tacrolimus can minimize the dose of tacrolimus in long-term stable LT patients.

## Data Availability Statement

The datasets generated for this study can be found in NCBI GEO, GEO Accession No. GSE161187.

## Ethics Statement

The studies involving human participants were reviewed and approved by the Institutional Review Board of the Catholic University of Korea. The patients/participants provided their written informed consent to participate in this study. The animal study was reviewed and approved by the Animal Care and Use Committee of the Catholic University of Korea.

## Author Contributions

SKL, M-JP, JYC, and M-LC designed the experiments. M-JP, JYJ, J-AB, JWC, and J-YR performed the experiments. SKL, M-JP, JWJ, SHB, SKY, HJC, YKY, JYC, and M-LC analyzed and interpreted the data. SKL, M-JP, JYC, and M-LC wrote the manuscript. JYC and M-LC supervised the study. All authors contributed to the article and approved the submitted version.

## Funding

This research was supported by a grant of the Korea Health Technology R&D Project through the Korea Health Industry Development Institute (KHIDI), funded by the Ministry of Health & Welfare, Republic of Korea (grant number: HI15C3062). This work was supported by the National Research Foundation of Korea (NRF) grant funded by the Korea government (MSIT) (No. 2020R1F1A1075816).

## Conflict of Interest

The authors declare that the research was conducted in the absence of any commercial or financial relationships that could be construed as a potential conflict of interest.

## Abbreviations

CNI, calcineurin inhibitor; LT, liver transplantation; IL-2, interleukin-2; Treg, regulatory T; Th17, type 17 helper; AMPK, 5′-AMP-activated protein kinase; mTOR, mammalian target of rapamycin; STAT3, signal transducer and activator of transcription 3; MMF, mycophenolate mofetil; GVHD, graft-versus-host disease; DM, diabetes mellitus; BMT, bone marrow transplant; IFN, interferon; KEGG, Kyoto encyclopedia of genes and genomics; WT, wild-type; ANOVA, one-way analysis of variance; Th1, type 1 helper T.
