# Supplementary material for: Combination Treatment With Metformin and Tacrolimus Improves Systemic Immune Cellular Homeostasis by Modulating Treg and Th17 Imbalance
Source: Front Immunol. 2021 Jan 8;11:581728. doi: 10.3389/fimmu.2020.581728 (PMC7821164; doi:10.3389/fimmu.2020.581728)
Supplement: Supplementary file 1 [file DataSheet_1.pdf]

Supplementary Material

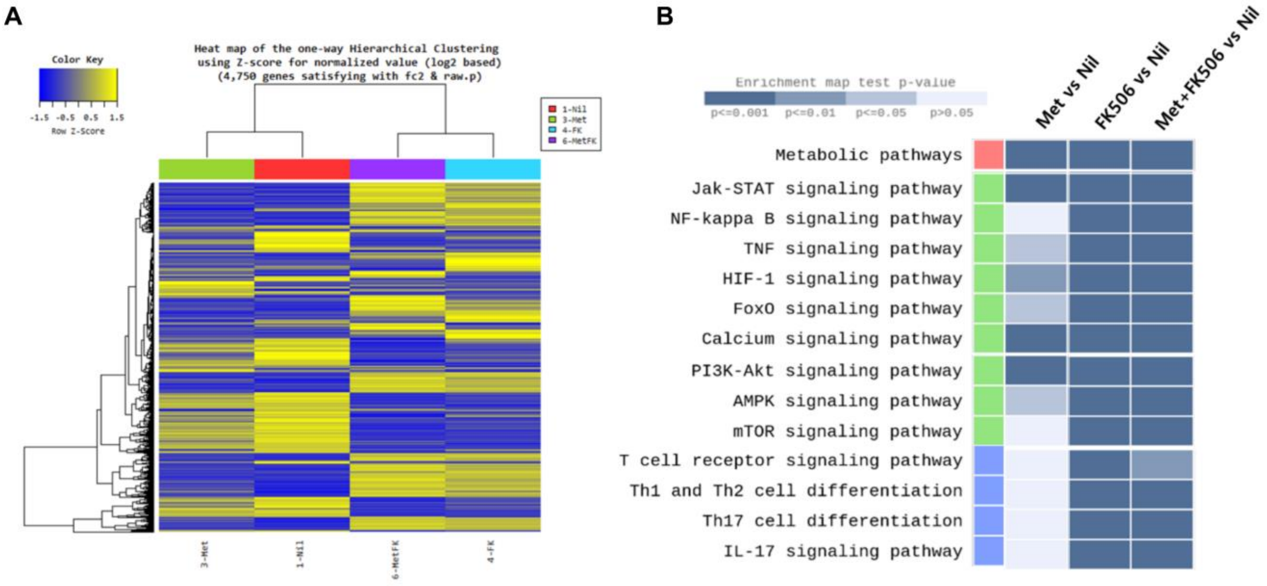

Supplementary Figure 1. Hierarchical clustering analysis and KEGG pathway analysis according to metformin or tacrolimus monotherapy and combination therapy. (A) Hierarchical clustering analysis of the patterns in the expression of 4,750 genes in control, metformin, tacrolimus and combination group. The horizontal axis refers to each treatment; the vertical axis refers to the difference between clusters. (B) KEGG pathway analysis of metabolic, Treg and Th17 signaling pathway in each treatments compared to control.

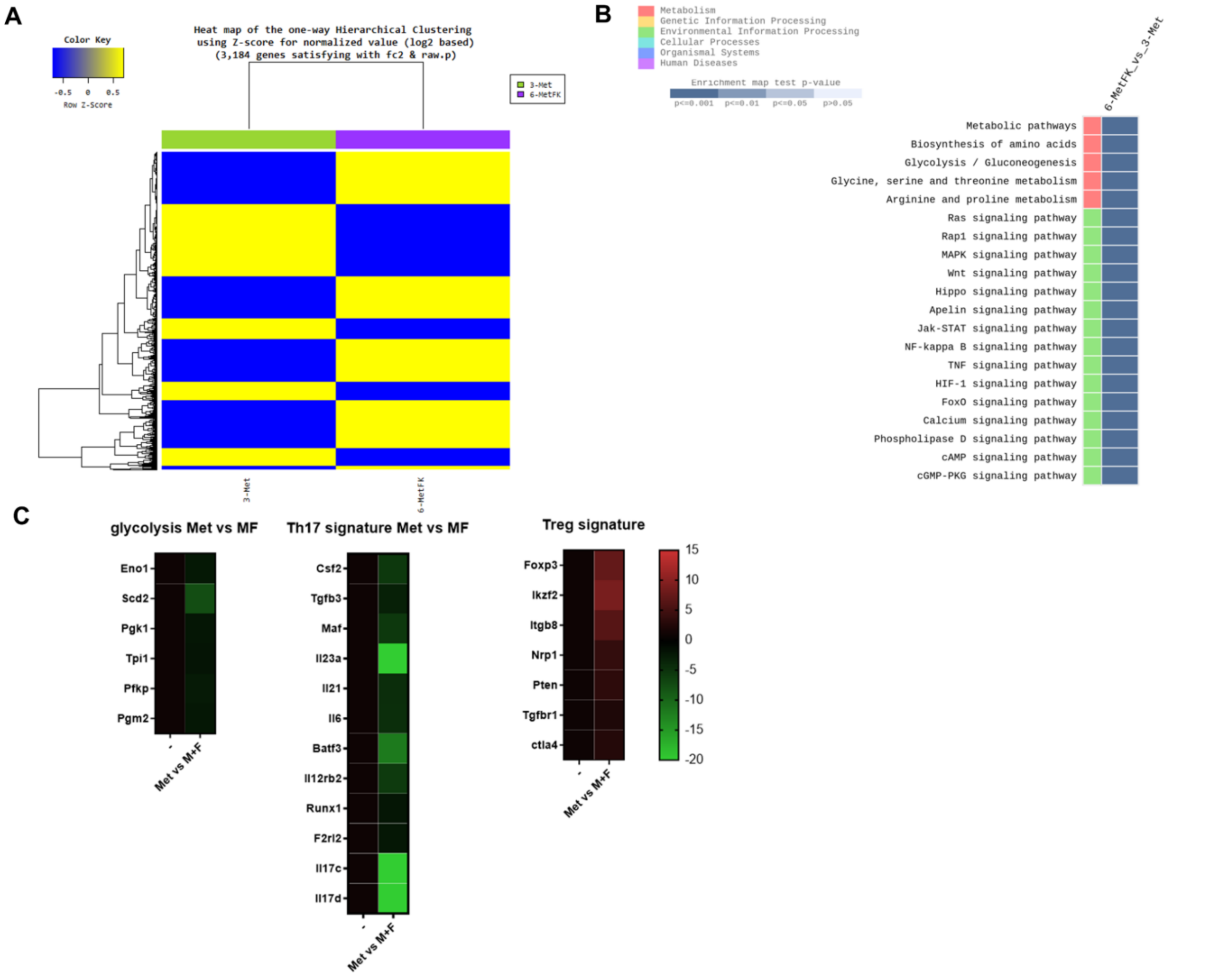

Supplementary Figure 2. Hierarchical clustering analysis, KEGG pathway analysis and RNA sequencing analysis comparing combination therapy to metformin monotherapy. (A) Hierarchical clustering analysis of the patterns in the expression of 3,184 genes in metformin and combination group. The horizontal axis corresponds to the treatment the vertical axis corresponds to the difference between clusters. (B) KEGG pathway analysis for metabolic, Treg and Th17 signaling pathway comparing metformin to combination therapy. (C) Gene expression analysis by RNA sequencing comparing metformin to combination therapy. Heatmaps of genes encoding for molecules involved in glycolysis, Th17 cells and Treg cell function that are differentially expressed in combination of metformin and FK506 compared to metformin.
